# The Importance of Integrating Clinical Relevance and Statistical Significance in the Assessment of Quality of Care –Illustrated Using the Swedish Stroke Register

**DOI:** 10.1371/journal.pone.0153082

**Published:** 2016-04-07

**Authors:** Anita Lindmark, Bart van Rompaye, Els Goetghebeur, Eva-Lotta Glader, Marie Eriksson

**Affiliations:** 1 Department of Statistics, Umeå School of Business and Economics, Umeå University, Umeå, Sweden; 2 Department of Applied Mathematics, Computer Science and Statistics, Ghent University, Ghent, Belgium; 3 Department of Public Health and Clinical Medicine, Umeå University, Umeå, Sweden; Stanford University School of Medicine, UNITED STATES

## Abstract

**Background:**

When profiling hospital performance, quality inicators are commonly evaluated through hospital-specific adjusted means with confidence intervals. When identifying deviations from a norm, large hospitals can have statistically significant results even for clinically irrelevant deviations while important deviations in small hospitals can remain undiscovered. We have used data from the Swedish Stroke Register (Riksstroke) to illustrate the properties of a benchmarking method that integrates considerations of both clinical relevance and level of statistical significance.

**Methods:**

The performance measure used was case-mix adjusted risk of death or dependency in activities of daily living within 3 months after stroke. A hospital was labeled as having outlying performance if its case-mix adjusted risk exceeded a benchmark value with a specified statistical confidence level. The benchmark was expressed relative to the population risk and should reflect the clinically relevant deviation that is to be detected. A simulation study based on Riksstroke patient data from 2008–2009 was performed to investigate the effect of the choice of the statistical confidence level and benchmark value on the diagnostic properties of the method.

**Results:**

Simulations were based on 18,309 patients in 76 hospitals. The widely used setting, comparing 95% confidence intervals to the national average, resulted in low sensitivity (0.252) and high specificity (0.991). There were large variations in sensitivity and specificity for different requirements of statistical confidence. Lowering statistical confidence improved sensitivity with a relatively smaller loss of specificity. Variations due to different benchmark values were smaller, especially for sensitivity. This allows the choice of a clinically relevant benchmark to be driven by clinical factors without major concerns about sufficiently reliable evidence.

**Conclusions:**

The study emphasizes the importance of combining clinical relevance and level of statistical confidence when profiling hospital performance. To guide the decision process a web-based tool that gives ROC-curves for different scenarios is provided.

## Introduction

Measuring and monitoring performance is an essential component of quality improvement strategies in health care. Assessments of hospital performance are widely used for both internal guidance and evaluation purposes. They provide a basis for decisions by policy makers, health care providers, and patients.

Several hospital performance and quality measurement systems have been implemented worldwide, such as the National Peer Review Programme by the National Health Services (NHS) in the UK [1], the World Health Organization’s Performance Assessment Tool for Quality Improvement in Hospitals (PATH) [2], and The Centers for Medicare & Medicaid Services’ Hospital Compare in the US [3]. In Sweden, a system of around 80 quality registers that are jointly financed by the central government and county councils has been built to monitor and improve the quality of care [4]. A majority of the registers provide publicly available comparisons of hospital quality-of-care parameters.

Several factors affect the precision of estimated hospital performance, including the choice of performance measure, patient volume, the patient case-mix and its modeled impact, plus simple random variation. Standard methods to report hospital performance include league tables, forest plots, and funnel plots, displaying the means of an outcome or a process indicator across hospitals. Their strengths and limitations have been extensively discussed [5–9]. Statistically significant deviations from a baseline such as the national average are typically seen as indicators of outlying performance. Important deviations are then difficult to detect in small hospitals, and in large hospitals such testing might point to outlying performance even for small deviations that are not clinically relevant. Therefore, consideration needs to be given to both the statistical properties of the decision criterion used to flag a hospital as outlying and to the practical significance of the deviations that are flagged. The prevalent approach; focusing on controlling the type I error rate to ensure that few hospitals are erroneously flagged, at the cost of lower power to detect outliers, may be appropriate for some situations but not for others. For decisions to have the desired impact, such as efficient allocation of resources, it is important that deviations that are flagged are clinically relevant.

Developments have been and continue to be made in the area of health care quality assessment, including the impact of using different types of performance measures [10, 11] and alternative statistical methods to address methodological issues such as small hospital size [12–15].

We examine the properties, including sensitivity and specificity, of a suggested benchmarking method [13, 15] that considers what constitutes a clinically relevant deviation along with the degree of statistical evidence required before labeling the deviation as relevant. This is done through a simulation study based on data from the Swedish Stroke Register (Riksstroke). We focus on the performance indicator death or dependence in activities of daily living (ADL) at 3 months after stroke, but the method is generally applicable to other outcomes and process indicators.

The objective of this study is to support this important but complicated decision process by highlighting the effect and relative importance of the choice of the benchmark value and required statistical evidence level on the results. An Excel tool available online is provided to guide the decision-making process in other settings.

## Data and Methods

### Data

The study was based on 18,309 patients registered in Riksstroke during the years 2008 and 2009. The patients included were 18–80 years old, were registered as having a first-time acute stroke, and were independent in ADL at the time of stroke. The main purpose of Riksstroke is to monitor and support improvement of quality of stroke care in Sweden [16]. It was established in 1994 and covers all hospitals in the country that admit acute stroke patients (76 hospitals in 2009). Information is collected during the acute phase and at follow-ups 3 months and 1 year after the stroke. In 2009, Riksstroke covered 85% of acute stroke cases, and just over 89% of those registered during the acute phase were followed up at 3 months. For more details, see the Riksstroke website http://www.riksstroke.org.

The performance indicator, dead or dependent in ADL at 3 months after stroke, was defined as the patient being registered as dependent in ADL at the 3 month follow-up or the patient dying within 3 months after stroke. Independence in ADL was defined as the patient being able to manage dressing, using the toilet, and walking indoors unassisted. Dates of death were retrieved for all patients from the Swedish Cause of Death Register managed by the National Board of Health and Welfare using the Swedish personal identification number. Thus patients with missing outcomes were those that were alive but did not provide follow-up information at 3 months after their stroke.

Baseline patient characteristics were used for case-mix adjustments. The clinically most important predictors of death or ADL dependency were included: age, sex, level of consciousness at admission, stroke subtype, smoking status, atrial fibrillation, and diabetes. The level of consciousness at admission to the hospital was used as a proxy for stroke severity. It was registered using three levels based on the Reaction Level Scale (RLS) [17] where fully conscious corresponds to RLS 1, drowsy to RLS 2–3, and unconscious to RLS 4–8. Stroke subtypes were intra-cerebral hemorrhagic (ICD-10 code I61), ischemic (I63), and unspecified stroke (I64). Smoking status was coded as Yes/No/Unknown due to a large proportion of missing information on baseline smoking status.

All patients and their next of kin are informed about registration in the quality register Riksstroke, that the aim of the register is to support high and consistent quality of care for stroke patients throughout Sweden, and that data may be used for developing and ensuring the quality of stroke treatment, for compiling statistics and for health care research purposes. They are informed of their rights to deny participation (opt-out consent). Consent is not collected for specific research projects in addition to this general consent. Participation in this project involves no direct risk for the patients. Data were de-identified prior to being received by the researchers. Riksstroke in general (reference number 95–168) and this project (212-321-31M) has been approved by the Regional Ethical Review Board in Umeå.

### Statistical method for case-mix adjustment

We used direct standardization to adjust the outcome for differences in patient case-mix over hospitals. A hospital-specific standardized risk of death or dependency, *R*_*h*_, then estimates the risk expected across the entire study population should all patients receive the level of care offered at hospital h. Each hospital is thus evaluated on a common reference population.

Several estimators exist for these hospital-specific risks, and the relative performance of different modelling approaches for case-mix adjustment, such as fixed versus random hospital effects, has been evaluated in the context of provider profiling.[13, 18, 19] Following recommendations in [15], we start from a fixed effects multiple logistic regression model, adding the hospital effects to effects of patient-specific covariates recorded at baseline:

$$\log\left(\frac{p_{i}}{1-p_{i}}\right)=\beta_{0}+X_{i}^{'}\beta +\sum_{h=2}^{m}I_{h}\cdot \psi_{h},$$ (1)

where *p*_*i*_ is the risk of death or dependence for patient *i* and β is the vector of regression parameters associated with the patient specific covariates *X*. *I*_*h*_ is an indicator variable that takes value 1 if patient *i* was treated in hospital *h* and 0 otherwise, and *ψ*_*h*_ is the hospital effect for hospital *h*. We assume that the hospital effects are constant regardless of the patient-specific covariate values and thus no hospital-covariate interactions are included in the model. It has been shown that our inference is robust against violations of this assumption (see discussion).

If the covariates in *X* are sufficient to adjust for case-mix, we can estimate *R*_*h*_ by replacing the sum in Eq 1 for all patients with the estimated hospital effect for hospital *h*, *ψ*_*h*_, regardless of where they were actually treated. Let us define the risk of patient *i* under the care level of hospital *h* as *p*_*ih*_. Suppose for simplicity that we need to adjust for age only. Logistic regression then models the risk of patient *i* being dead or dependent at 3 months under the potential care level of hospital *h* as:

$$\log\left(\frac{p_{ih}}{\left(1-p_{ih}\right)}\right)=\beta_{0}+\beta_{1}(\text{Age of patient i})+\psi_{h},$$ (2)

where β_1_ is the increase in log-odds per one-year increase in age. Note that the hospital effect has been fixed to *ψ*_*h*_ regardless of where patient *i* was actually treated.

The standardized risk for hospital *h*, *R*_*h*_, is then the average of *p*_*ih*_ over all *n* patients. This standardized risk can be interpreted as the estimated proportion of patients being dead or dependent if all had received the same quality of care as that of hospital *h*. For a more formal description of the standardization, see section 1 of S1 Appendix.

### Decision criterion

Quality control is often concerned with detecting underperforming hospitals. For our outcome this means a focus on high outliers, and we aim to diagnose hospital-specific risks exceeding the current population risk by a meaningful margin. Consensus among the clinical expertize should be the basis for the maximum tolerated value of the hospital-specific risk *R*_*h*_. This value will further depend on the study purpose and foreseeable implications of the hospitals’ screening results. An early warning signal privately delivered to the hospital should be less conservative than a formally published diagnosis of the hospital crossing the benchmark. For our scientific work we chose to express the benchmark risk through a parameter *δ*, the relative increase of the current population risk. We thus have (1 + *δ*) times the observed population risk as the benchmark for *R*_*h*_. For example, a hospital-specific standardized risk may be clinically acceptable as long as it does not exceed the population risk by more than 20%. With *δ* = 0.20 and an observed population risk of 25% the benchmark becomes (1.20 × 0.25), corresponding to a 30% hospital-specific standardized risk.

Identifying hospitals with excess risk from a sample of patients needs a decision process that accounts for sampling variation and resulting uncertainty. Available statistical information is commonly summarized through the confidence interval for the standardized risk. Accordingly, hospital *h* may be classified as outlying when we are *k* x 100% certain that the true hospital-specific standardized risk *R*_*h*_, exceeds the benchmark value. [12, 13, 15] Technically we then check whether the (lower bound) of the *k* × 100% one-sided confidence interval for *R*_*h*_ exceeds (1 + *δ*) times the observed population risk. Calculation of this CI can be based on a normal approximation, as shown by Varewyck, Goetghebeur (15]. Part 2 of S1 Appendix describes the derivation of the variance of the estimated *R*_*h*_.

For a given benchmark (a given *δ*), patient case-mix and risk setting, the chosen confidence level *k* will determine the sensitivity and specificity of our hospital diagnoses.

### Simulations

The properties of the proposed method were investigated through a simulation study mimicking the data structure found in Riksstroke. The model (1) fitted to the original Riksstroke data associates a predicted risk with each patient’s covariate values. We randomly generated independent binary outcomes following these risks to create 1000 new simulated data sets.

Next, we analyzed each simulated data set as if it were the original one. From its observed population risk we found the simulation-specific benchmark. After refitting the logistic regression model we derived the corresponding standardized risk for hospital *h*, *R*_*sh*_, from each simulated data set s. When its *k* × 100% one-sided CI exceeded the benchmark, hospital *h* was labeled with excess risk for simulation s. Otherwise the hospital was labeled as having acceptable performance. So for a chosen confidence level *k* and a given margin of excess risk *δ*, 1000 diagnoses were derived for each of the 76 hospitals. The simulation algorithm is outlined in section 3 of the S1 Appendix.

The 1000 simulated classifications were then compared to the gold standard, the classifications obtained from the original Riksstroke data. A hospital *h* was classified as having true excess risk in the simulated setting when its standardized risk *R*_*h*_ from the original logistic regression fit exceeded the benchmark, (1 + *δ*) times the originally observed population risk.

We could thus calculate the percentage correctly classified hospitals over all simulated data sets as well as sensitivity, specificity, and positive and negative predictive values (PPV and NPV, respectively) for the decision process. This was done for selected levels of confidence, with *k* ranging from 0.01 (low) to 0.99 (high) and for benchmark values corresponding to *δ* = 0, 0.10, 0.15, and 0.20. Benchmarks hence varied from the observed population risk to a 20% relative increase.

Simulations and analyses were performed using the R 2.15.2 software environment (The R Foundation for Statistical Computing).

## Results

### Original data

Of the 21,376 patients that met the inclusion criteria, 2,788 (13.0%) were lost to follow-up at 3 months, and another 279 (1.3%) had missing information for one or more covariates other than smoking status. The group that lacked data on the 3 month follow-up was on average younger and included a slightly larger proportion of patients with hemorrhagic stroke, a larger proportion of smokers, and a smaller proportion of patients with atrial fibrillation (see Table 1). For the purpose of our illustration here, cases with missing data, except for the large patient group with missing smoking status, were deleted, leaving a total of 18,309 patients in the study.

**Table 1:** Patient characteristics grouped by availibity of three month follow-up.

	With follow-up (n = 18,309)		Lost to follow-up (n = 2,788)	
Covariate	Mean/Proportion	Standard error	Mean/Proportion	Standard error
*Age (Years)*	67.6	0.07	64.7	0.23
*Male sex*	58.9%	0.36%	61.0%	0.92%
*Level of consciousness*				
*Alert*	87.8%	0.24%	87.1%	0.64%
*Drowsy*	8.3%	0.20%	9.9%	0.57%
*Unconscious*	3.9%	0.14%	3.0%	0.32%
*Subtype*				
*Hemorrhagic*	13.1%	0.25%	15.5%	0.69%
*Ischemic*	84.9%	0.26%	81.9%	0.73%
*Unspecified*	2.0%	0.10%	2.6%	0.30%
*Smoking*				
*No*	72.5%	0.33%	63.8%	0.91%
*Yes*	20.0%	0.30%	23.7%	0.81%
*No information*	7.4%	0.19%	12.5%	0.63%
*Atrial fibrillation*	18.2%	0.29%	16.2%	0.70%
*Diabetes*	19.6%	0.29%	19.2%	0.75%

**Fig 1:**
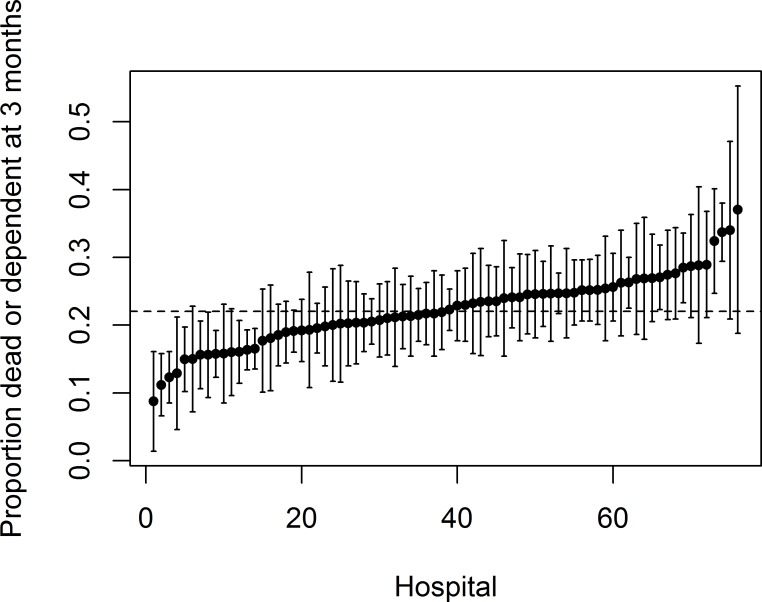
Caterpillar plot of the proportion dead or dependent 3 months after stroke with 95% confidence intervals for each hospital. The dashed line represents the observed population risk (based on 18,309 patients).

Time from stroke to the scheduled ‘3 month’ follow-up varied with a median of 91 days and 95% of the follow-ups occurred between 74 and 153 days. All these follow-up data were included. At the 3 month follow-up 22.0% of patients (95% CI: 21.4–22.6%) had died or were dependent in ADL. Over the 76 hospitals, the median number of patients was 194 (range 27–798 patients) with observed risks of death or dependency ranging from 8.8% to 37.0%, as illustrated by the caterpillar plot in Fig 1.

Most hospitals had a similar case-mix with the exception of a few extreme hospitals (see Table 2). For example, the average patient ages among the hospitals had a median of 68.4 years, but the lowest average age for an individual hospital was 60.6 years. The proportion of patients who were fully conscious at arrival ranged from 73.3% to 95.1%.

**Table 2:** Patient characteristics (means and proportions) at the hospital level.

Covariate	Min	Q1	Median	Q3	Max
*Dead or dependent at 3 months*	8.8%	19.2%	22.1%	25.2%	37.0%
*Age (Years)*	60.6	67.3	68.4	69.0	72.6
*Male sex*	50.6%	56.8%	58.5%	61.5%	69.5%
*Level of consciousness*					
*Alert*	73.3%	85.4%	88.6%	91.1%	95.1%
*Drowsy*	3.0%	6.0%	7.9%	10.3%	18.3%
*Unconscious*	0.0%	2.2%	3.5%	4.9%	13.3%
*Subtype*					
*Hemorrhagic*	5.1%	10.1%	12.7%	15.1%	27.0%
*Ischemic*	64.6%	82.2%	85.2%	88.4%	94.9%
*Unspecified*	0%	0%	1.2%	2.9%	27.2%
*Smoking*					
*No*	48.1%	68.7%	73.3%	77.7%	84.2%
*Yes*	12.0%	16.0%	19.1%	21.3%	31.6%
*No information*	0.0%	3.5%	6.1%	11.9%	37.0%
*Atrial fibrillation*	11.6%	16.5%	18.3%	20.0%	31.2%
*Diabetes*	10.5%	17.7%	19.8%	22.4%	31.2%

**Fig 2:**
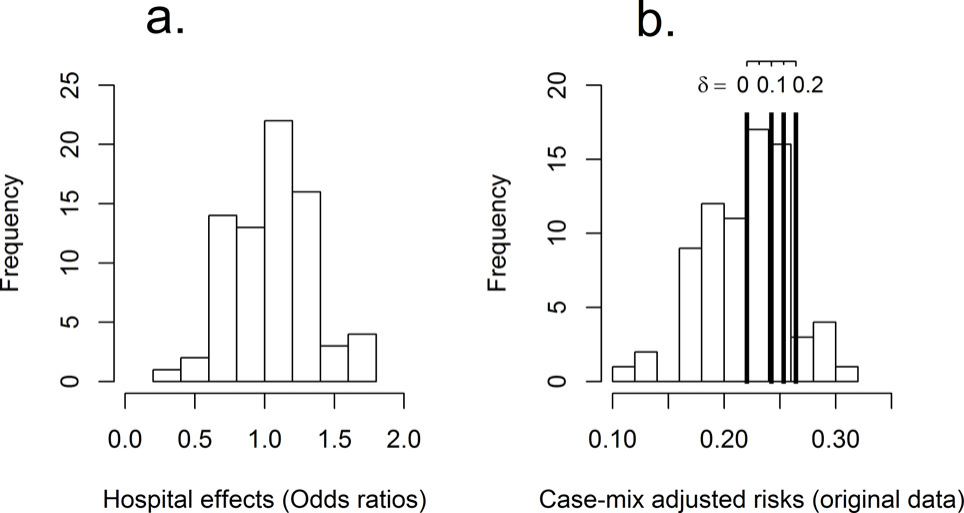
a: Hospital effects (odds ratios) from the logistic regression model (each individual hospital compared to the average over all hospitals). b: Standardized risks (original data) with lines for the benchmark values ((1 + δ) observed population risk) for different values of δ.

Multiple logistic regression showed that older age, female sex, lower level of consciousness at admission, atrial fibrillation, diabetes, smoking, and hemorrhagic stroke were associated with significantly higher risk of death or dependence (Table 3). The estimated odds ratios comparing individual hospital risks to the average over all hospitals after adjustment for case-mix (i.e. using effects coding) ranged from 0.3 to 1.8 with a majority in the 0.8–1.2 range (Fig 2A).

**Table 3:** Estimated effects from multiple logistic regression modeling death or dependency at 3 months. Presented with estimated standard errors (SE), odds ratios (OR) and confidence intervals (CI).

	Estimated	SE	Estimated OR	95% CI	P-value
	β			of OR	
*Intercept*	−4.495	0.183	0.011	[0.008; 0.016]	<0.001
*Age*	0.048	0.002	1.050	[1.045; 1.055]	<0.001
*Sex*					0.016
*Female*	Reference				
*Male*	−0.101	0.042	0.904	[0.833; 0.981]	
*Level of consciousness at admission*					<0.001
*Alert*	Reference				
*Drowsy*	2.075	0.061	7.964	[7.060; 8.984]	
*Unconscious*	3.336	0.115	28.108	[22.441; 35.207]	
*Type of stroke*					<0.001
*Hemorrhagic*	Reference				
*Ischemic*	−0.870	0.057	0.419	[0.375; 0.469]	
*Unspecified*	−1.078	0.167	0.340	[0.245; 0.472]	
*Smoking*					<0.001
*No*	Reference				
*Yes*	0.162	0.055	1.176	[1.056; 1.309]	
*No information*	0.551	0.074	1.734	[1.501; 2.004]	
*Atrial fibrillation*					<0.001
*No*	Reference				
*Yes*	0.378	0.050	1.459	[1.323; 1.609]	
*Diabetes*					<0.001
*No*	Reference				
*Yes*	0.422	0.049	1.525	[1.385; 1.678]	

Comparing hospital-specific risks to the (1 + δ) times (observed population risk) benchmark lead to 40 (22, 12, and 7, respectively) out of the 76 hospitals exceeding the benchmark for *δ* = 0 (0.10, 0.15, 0.20), i.e. 22.0% (24.2%, 25.3%, 26.4%) risk at 3 months. Fig 2B shows a histogram of the hospital-specific standardized risks with lines marking the different benchmark values.

### Diagnostic properties of the method for identifying outlying performance

**Fig 3:**
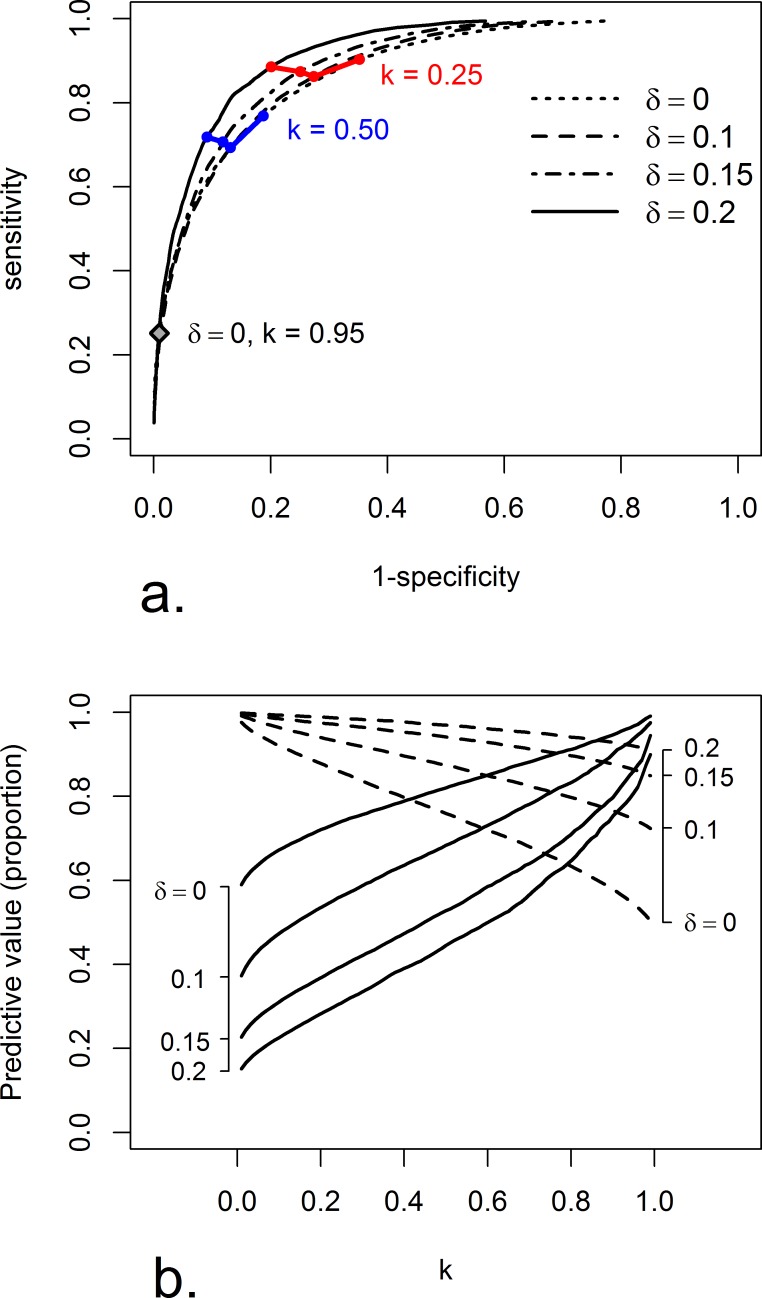
a: ROC curves for different values of δ and k based on 1000 simulations. b: Positive (solid lines) and negative (dashed lines) predictive values for different values of k and δ based on 1000 simulations.

Sensitivity and specificity reflect the method’s ability to correctly classify hospitals with excess risk or acceptable levels of care. Table 4 shows results for the different benchmarks (δ) and selected levels of statistical confidence (k). These are summarized in ROC curves in Fig 3A. For a given benchmark value (i.e. a given *δ*), demanding more evidence (*k*) before labeling a hospital as ‘outlying’ naturally leads to fewer positive decisions irrespective of the truth and therefore lower sensitivity and increased specificity.

**Table 4:** Sensitivity and specificity for different values of acceptable deviation (δ) and statistical evidence (k).

	δ = 0		δ = 0.1		δ = 0.15		δ = 0.2	
k × 100 (%)	*Sens*.	*Spec*.	*Sens*.	*Spec*.	*Sens*.	*Spec*.	*Sens*.	*Spec*.
10	0.963	0.478	0.948	0.570	0.950	0.604	0.957	0.667
30	0.879	0.686	0.835	0.761	0.846	0.782	0.857	0.827
50	0.769	0.813	0.693	0.869	0.707	0.881	0.718	0.909
75	0.565	0.928	0.453	0.956	0.463	0.958	0.471	0.970
90	0.359	0.977	0.257	0.988	0.259	0.988	0.244	0.992
99	0.112	0.999	0.062	0.999	0.053	0.999	0.038	1.000

On the other hand, seeking to diagnose fewer hospitals with more severe levels of excess risk, i.e. increasing *δ* while keeping the demanded confidence level *k* fixed, has less obvious implications for the diagnostic properties of our procedure. Sensitivity depends on the distribution of the hospital-specific risks above the benchmark, shown in Fig 2B. Hospitals which exceed the benchmark with a greater margin will be easier to detect, even if they are relatively small in size. In our case, a higher *δ* led to little change in sensitivity (in either direction) while specificity tended to increase. This meant the ROC curve hardly changed in height (sensitivity) but shifted to the left (changing specificity) as illustrated for *k* = 0.25 and *k* = 0.5 in Fig 3A. The stability of the sensitivity indicates that concerns about sufficiently reliable evidence should not influence the choice of a clinically relevant benchmark.

For a given benchmark value (*δ*) the positive predictive value, PPV, increased and the negative predictive value, NPV, decreased with increasing *k* (Fig 3B.). Because more evidence was required to be labeled ‘outlying’, the risk of being wrongly classified as such decreased and, conversely, the risk of being wrongly classified as ‘acceptable’ increased.

Again, determining the choice of *k* that gives a desired PPV or NPV is not straightforward, and this choice is affected by the distribution of the standardized hospital risks in the observed hospital population. In our setting, increasing *δ* (while keeping *k* fixed) decreased the PPV and increased the NPV (Fig 3B.). A higher benchmark leads to a larger number of truly ‘acceptable’ hospitals that run the risk of being wrongly classified as ‘outlying’ leading in turn to a tendency for a lower PPV.

As a concluding example, using the “standard” method with 95% confidence compared to the overall average (*k* = 0.95 and *δ* = 0) gave a sensitivity of 0.252 and a specificity of 0.991 (marked with a diamond in Fig 3A.). That is, the accuracy of identifying ‘acceptable’ performers was high, but the accuracy of identifying ‘outlying’ performers was quite low. By choosing *k* = 0.5, we obtained a sensitivity of 0.769 and a specificity of 0.813 meaning a relatively high accuracy in identifying both types of hospitals. Focusing on the predictive values, the standard method gave PPV = 0.967 and NPV = 0.544. This means that a classification as ‘outlying’ is likely to reflect the true performance of the hospital, while an ‘acceptable’ classification only reflects the true state of the hospital in just over half of all cases. A choice of *k* = 0.5 gave PPV = 0.820 and NPV = 0.760.

## Discussion

We have illustrated the characteristics of a method for benchmarking hospital performance that integrates statistical evidence with what is considered to be a clinically relevant deviation. With the standard method, comparing 95% confidence intervals to the overall average, less than 1% of hospitals with acceptable performance would be erroneously flagged (high specificity). However, in this scenario, 75% of hospitals with outlying performance would remain undetected (low sensitivity). Decreasing the level of statistical evidence required to label a hospital as having outlying performance had the expected effects of increasing sensitivity and decreasing specificity. The effect of choosing a higher benchmark value was less obviously anticipated. Sensitivity is the ratio of the number of correctly identified outliers to the total number of outlying hospitals and both the numerator and denominator change with the benchmark value. These dynamics are affected by the shape of the upper tail of the distribution of the standardized risks. Specificity was not affected in the same way by these dynamics, and it increased predictably with increasing benchmark values. This is, however, not automatically the case. For example, one might simultaneously be interested in identifying hospitals that perform better than expected, which would give three-way classifications. Then the lower tail of the distribution of standardized risks would also have an effect.

General recommendations regarding an optimal choice of level of evidence *k* cannot be provided because these are specific to the purpose of the comparison and the quality indicator. To help with these decisions, we have provided an Excel tool “Web tool ROC” (available at http://www.riksstroke.org/software/). In this tool, the user can insert relevant hospital risks (e.g. the standardized risks with their standard errors from the previous year) and select a benchmark by providing a value of *δ*. The theoretical sensitivities and specificities that result from different values of statistical confidence are then calculated and summarized through ROC curves.

The Swedish National Board of Health and Welfare profiles health care performance based on rankings according to a hospital’s position relative to others (low, mid, and top tertile) [20]. The NHS uses target levels,[1] and Riksstroke uses objectively defined values for acceptable and high performance [21]. Here, we used a benchmark that is relative to the average national hospital performance and includes a clinically relevant deviation. Hence, the drawback with relative rankings that always identify the same number of hospitals regardless of the general level of performance is avoided, and at the same time the general level of care is accounted for and hence allowed to change over time as new treatments become available or the population grows older. An added advantage is that the flexibility of the benchmark allows a change in setting, e.g., to a specific disease subtype or to a subgroup of the population. The decision of the benchmark is made by clinicians, e.g., through Delphi processes and consensus groups.

The hospitals in our application had similar case-mix, and for an acute illness such as stroke it can be argued that all hospitals should be able to treat the same type of patients rather than being specialized for a certain patient group. Therefore, adjustment for case-mix was performed using direct standardization by comparing hospitals based on the entire study population rather than indirect standardization where hospitals are evaluated based on their own patients. This facilitates straightforward national comparisons and joint benchmark values for policy makers because hospitals are evaluated against the same reference population, and the potential lack of comparability with non-overlapping case-mix in indirect standardization is avoided [22, 23]. In a different set-up a parallel development may be followed for indirect standardization.

The logistic regression model used here was kept simple, since the goal of the paper is to illustrate the method and not to make inferences about this particular outcome. The model could be extended to include additional covariates and interactions, e. g., hospital-covariate interactions to allow the hospital effects to vary across covariate values. The assumption of constant hospital effects would be violated e.g. when some hospitals are better than others at treating geriatric patients. Reassuringly, [24] showed that ignoring an existing hospital interaction has negligible impact when, as in our setting, the variation in patient case-mix is mild. For situations with small hospitals more sophisticated modelling approaches could be applied to avoid bias [15]. Variables that are important to include in case-mix adjustment of stroke outcome for hospital comparisons have recently been suggested [25], and an important variable is stroke severity [25, 26]. Our study included consciousness at hospital admission as a proxy for stroke severity and for example did not include prior myocardial infarction or hyperlipidemia, which might cause residual confounding.

When deciding on a benchmarking approach it is important to take into account the purpose of the benchmarking. Will the results be used for internal or external purposes? Is it more important to have a procedure with low chance of missing outlying hospitals (high sensitivity) than one with low chance of wrongly classifying hospitals as outlying (high specificity)? The “standard” method with 95% confidence compared to the overall average seems to be useful only in the latter setting, having a very high specificity at the cost of missing a large portion of hospitals with outlying performance. With a focus on predictive values we want to know whether a classification as having outlying/acceptable performance is likely to reflect the true performance of the hospital. These considerations affect the choice of the optimal statistical confidence level. The aforementioned web-based tool can be used to guide policy makers when making the necessary choices.

## Conclusions

We have emphasized the importance of integrating clinical and statistical evidence when evaluating hospital performance and have studied the properties of a targeted method that achieves this goal in the context of the Swedish Stroke Register. The statistical confidence level used affects the balance between sensitivity and specificity depending on which deviation we seek to discover in the hospital population. A good choice will account for the purpose of the benchmarking and the context in which the results of the benchmarking will be used.

## Supporting Information

S1 Appendix Theory and simulations. (DOCX)

## References

[1] NHS Improving Quality. National peer review programme 2014 [cited 2015 November 10]. Available from: http://www.nationalpeerreview.nhs.uk/.

[2] World Health Organization. Performance assessment tool for quality improvement in hospitals 2007 [cited 2015 November 10]. Available from: http://www.pathqualityproject.eu/.

[3] Centers for Medicare and Medicaid Services. Hospital Compare. 2014 [cited 2015 November 10]. Available from: https://www.cms.gov/Medicare/Quality-Initiatives-Patient-Assessment-Instruments/HospitalQualityInits/HospitalCompare.html.

[4] National Board of Health and Welfare, Swedish Association of Local Authorities and Regions. Översyn av de nationella kvalitetsregistren, guldgruvan i hälso och sjukvården Förslag till gemensam satsning 2011–2015. Stockholm: Ljungbergs tryckeri; 2010.

[5] van Dishoeck AM, Looman CW, van der Wilden-van Lier EC, Mackenbach JP, Steyerberg EW. Displaying random variation in comparing hospital performance. BMJ quality & safety. 2011;20(8):651–7. Epub 2011/01/14. 10.1136/bmjqs.2009.035881 . 21228432

[6] Goldstein H, Spiegelhalter DJ. League tables and their limitations: Statistical issues in comparisons of institutional performance. J R Stat Soc a Stat. 1996;159:385–409. 10.2307/2983325 .

[7] Spiegelhalter DJ. Funnel plots for comparing institutional performance. Statistics in medicine. 2005;24(8):1185–202. Epub 2004/11/30. 10.1002/sim.1970 . 15568194

[8] Kunadian B, Dunning J, Roberts AP, Morley R, de Belder MA. Funnel plots for comparing performance of PCI performing hospitals and cardiologists: demonstration of utility using the New York hospital mortality data. Catheterization and cardiovascular interventions: official journal of the Society for Cardiac Angiography & Interventions. 2009;73(5):589–94. Epub 2009/03/25. 10.1002/ccd.21893 . 19309714

[9] Schulman J, Spiegelhalter DJ, Parry G. How to interpret your dot: decoding the message of clinical performance indicators. Journal of perinatology: official journal of the California Perinatal Association. 2008;28(9):588–96. Epub 2008/07/18. 10.1038/jp.2008.67 . 18633418

[10] Hubbard RA, Benjamin-Johnson R, Onega T, Smith-Bindman R, Zhu W, Fenton JJ. Classification accuracy of claims-based methods for identifying providers failing to meet performance targets. Statistics in medicine. 2014 10.1002/sim.6318 . 25302935 PMC4262572

[11] Kolfschoten NE, Marang-van de Mheen PJ, Wouters MW, Eddes EH, Tollenaar RA, Stijnen T, et al A combined measure of procedural volume and outcome to assess hospital quality of colorectal cancer surgery, a secondary analysis of clinical audit data. PloS one. 2014;9(2):e88737 10.1371/journal.pone.0088737 24558418 PMC3928280

[12] Austin PC, Naylor CD, Tu JV. A comparison of a Bayesian vs. a frequentist method for profiling hospital performance. J Eval Clin Pract. 2001;7(1):35–45. 10.1046/j.1365-2753.2001.00261.x . 11240838

[13] Normand SLT, Glickman ME, Gatsonis CA. Statistical methods for profiling providers of medical care: Issues and applications. J Am Stat Assoc. 1997;92(439):803–14. 10.2307/2965545 .

[14] Paddock SM. Statistical benchmarks for health care provider performance assessment: a comparison of standard approaches to a hierarchical Bayesian histogram-based method. Health services research. 2014;49(3):1056–73. 10.1111/1475-6773.12149 . 24461071 PMC4231585

[15] Varewyck M, Goetghebeur E, Eriksson M, Vansteelandt S. On shrinkage and model extrapolation in the evaluation of clinical center performance. Biostatistics. 2014;15(4):651–64. 10.1093/biostatistics/kxu019 24812420 PMC4173104

[16] Asplund K, Hulter Asberg K, Appelros P, Bjarne D, Eriksson M, Johansson A, et al The Riks-Stroke story: building a sustainable national register for quality assessment of stroke care. International Journal of Stroke. 2011;6(2):99–108. Epub 23 December 2010. 10.1111/j.1747-4949.2010.00557.x . 21371269

[17] Starmark JE, Stalhammar D, Holmgren E. The Reaction Level Scale (RLS85). Manual and guidelines. Acta Neurochir (Wien). 1988;91(1–2):12–20. Epub 1988/01/01. . 3394542 10.1007/BF01400521

[18] DeLong ER, Peterson ED, DeLong DM, Muhlbaier LH, Hackett S, Mark DB. Comparing risk-adjustment methods for provider profiling. Statistics in medicine. 1997;16(23):2645–64. . 9421867 10.1002/(sici)1097-0258(19971215)16:23<2645::aid-sim696>3.0.co;2-d

[19] Austin PC, Alter DA, Tu JV. The use of fixed- and random-effects models for classifying hospitals as mortality outliers: a Monte Carlo assessment. Medical decision making: an international journal of the Society for Medical Decision Making. 2003;23(6):526–39. 10.1177/0272989X03258443 . 14672113

[20] National Board of Health and Welfare, Swedish Association of Local Authorities and Regions. Öppna Jämförelser 2013 Västerås, Sweden: Socialstyrelsen Sveriges Kommuner och Landsting; 2013 [cited 2015 November 10]. Available from: http://www.socialstyrelsen.se/Lists/Artikelkatalog/Attachments/19238/2013-12-1.pdf.

[21] Riksstroke. Riks-Stroke Årsrapport 2012 Umeå: Riks-Stroke; 2013 [cited 2015 November 10]. Available from: http://www.riksstroke.org/wp-content/uploads/2014/02/Riks-Strokes_Arsrapport-2012.pdf.

[22] Shahian DM, Normand SL. Comparison of "risk-adjusted" hospital outcomes. Circulation. 2008;117(15):1955–63. Epub 7 April 2008. 10.1161/CIRCULATIONAHA.107.747873 . 18391106

[23] Shahian DM, Normand SL. What is a performance outlier? BMJ quality & safety. 2015;24(2):95–9. 10.1136/bmjqs-2015-003934 . 25605952

[24] Varewyck M, Vansteelandt S, Eriksson M, Goetghebeur E. On the practice of ignoring center-patient interactions in evaluating hospital performance. Statistics in medicine. 2015 10.1002/sim.6634 . 26303843 PMC5049670

[25] Katzan IL, Spertus J, Bettger JP, Bravata DM, Reeves MJ, Smith EE, et al Risk adjustment of ischemic stroke outcomes for comparing hospital performance: a statement for healthcare professionals from the american heart association/american stroke association. Stroke. 2014;45(3):918–44. Epub 25 January 2014. 10.1161/01.str.0000441948.35804.77 . 24457296

[26] Fonarow GC, Pan W, Saver JL, Smith EE, Reeves MJ, Broderick JP, et al Comparison of 30-day mortality models for profiling hospital performance in acute ischemic stroke with vs without adjustment for stroke severity. JAMA: the Journal of the American Medical Association. 2012;308(3):257–64. Epub 17 July 2012. 10.1001/jama.2012.7870 . 22797643

