# Supplementary material for: The Importance of Integrating Clinical Relevance and Statistical Significance in the Assessment of Quality of Care –Illustrated Using the Swedish Stroke Register
Source: PLoS One. 2016 Apr 7;11(4):e0153082. doi: 10.1371/journal.pone.0153082 (PMC4824466; doi:10.1371/journal.pone.0153082)
Supplement: S1 Appendix — (DOCX) [file pone.0153082.s001.docx]

**Appendix S1: Theory and simulations**

**1. Direct standardization**

Let *Y(h)* denote the outcome that would have been observed under the level of care of hospital *h (h=1,…,m)*. *R_h_* is then defined as the expected value of these potential outcomes in the observed population$R_{h}=E\left(Y\left(h\right)\right)$.

To estimate this we have used a logistic regression model, modelling the risk of being dead or dependent in ADL 3 months after stroke adjusting for case-mix, and including indicators for hospital choice:

$$\text{logit}\left(E\left(Y|\mathbf{L}\right)\right)=\mathbf{L}'\mathbf{\theta}=\mathbf{X}'\mathbf{\beta}+\sum_{h=2}^{m} \psi_{h}I\left(H=h\right)$$

where $Y$ is the observed outcome, **L** consists of all patient specific covariates including the hospital where the patient was actually treated and **θ** is the full set of model parameters. **β** is the vector of regression parameters associated with the patient specific covariates **X** and *ψ_h_* is the hospital effect for hospital *h*.

Assuming that **X** contains all possible confounders of hospital-outcome relationship we can then estimate *R_h_* by replacing the hospital effect of the hospital where the patient was actually treated with that of hospital *h* and averaging over all *n* observed patients:

$\hat{R}_{h}=\hat{E}\left(Y\left(h\right)\right)=\frac{1}{n}\sum_{i=1}^{n} \text{expit}\left(\mathbf{X}_{i}^{'}\hat{\mathbf{\beta}}+\hat{\psi}_{h}\right)$,

where

$$\text{expit}\left(\mathbf{X}_{i}^{'}\hat{\mathbf{\beta}}+\hat{\psi}_{h}\right)=\frac{\text{exp}\left(\mathbf{X}_{i}^{'}\hat{\mathbf{\beta}}+\hat{\psi}_{h}\right)}{1+\text{exp}\left(\mathbf{X}_{i}^{'}\hat{\mathbf{\beta}}+\hat{\psi}_{h}\right)}$$

**2. Asymptotic variance**

The derivation of the asymptotic variance of $\hat{R}_{h}$, denoted $\sigma_{h}^{2}$, is based on [Varewyck et al. (2014](#_ENREF_1)). First we perform a Taylor expansion of

$$\hat{R}_{h}=\frac{1}{n}\sum_{i=1}^{n} \text{expit}\left(\mathbf{X}_{i}^{'}\hat{\mathbf{\beta}}+\hat{\psi}_{h}\right)=\frac{1}{n}\sum_{i=1}^{n} U_{h}\left(\hat{\mathbf{\theta}},\mathbf{L}_{i}\right)$$

around the true parameter values $\mathbf{\theta}=\left(\mathbf{\beta}',\mathbf{\psi}'\right)$, where $\mathbf{\psi}=\left(\psi_{2},\ldots,\psi_{m}\right)$:

$\sqrt{n}\hat{R}_{h}=\frac{1}{\sqrt{n}}\sum_{i=1}^{n} U_{h}\left(\hat{\mathbf{\theta}},\mathbf{L}_{i}\right)=\frac{1}{\sqrt{n}}\sum_{i=1}^{n} U_{h}\left(\mathbf{\theta},\mathbf{L}_{i}\right)+\frac{\partial}{\partial\mathbf{\theta}}\left(\frac{1}{n}\sum_{i=1}^{n} U_{h}\left(\mathbf{\theta},\mathbf{L}_{i}\right)\right)\sqrt{n}\left(\hat{\mathbf{\theta}}-\mathbf{\theta}\right)+o_{p}(1)$. (1)

By noting that the MLE of **θ** satisfies the following set of estimating equations

$\frac{1}{n}\sum_{i=1}^{n} \mathbf{L}_{i}\left\{Y_{i}-U\left(\hat{\mathbf{\theta}},\mathbf{L}_{i}\right)\right\}=0$,

and performing a Taylor expansion of these around **θ** we get that

$\sqrt{n}\left(\hat{\mathbf{\theta}}-\mathbf{\theta}\right)=\frac{1}{\sqrt{n}}\sum_{i=1}^{n} A^{-1}S_{i}+o_{p}(1)$, (2)

Where

$S_{i}=\mathbf{L}_{i}\left\{Y_{i}-U\left(\hat{\mathbf{\theta}},\mathbf{L}_{i}\right)\right\}$,

and

$A=\frac{1}{n}\sum_{i=1}^{n} \mathbf{L}_{i}\frac{\partial U\left(\mathbf{\theta},\mathbf{L}_{i}\right)}{\partial\mathbf{\theta}}\approx E\left(\frac{\partial S_{i}}{\partial\mathbf{\theta}}\right)=\bar{A}$.

Combining (1) and (2) gives

$\sqrt{n}\hat{R}_{h}=\frac{1}{\sqrt{n}}\sum_{i=1}^{n} U_{h}\left(\mathbf{\theta},\mathbf{L}_{i}\right)+\frac{1}{n}\sum_{i=1}^{n} \left(\frac{\partial U_{h}\left(\mathbf{\theta},\mathbf{L}_{i}\right)}{\partial\mathbf{\theta}}\right)\frac{1}{\sqrt{n}}\sum_{i=1}^{n} \bar{A}^{-1}S_{i}+o_{p}(1)$.

So, finally

$$\begin{matrix} \sigma_{h}^{2}=Var\left(\hat{R}_{h}\right)\approx\frac{1}{n}Var\left(\sum_{i=1}^{n} U_{h}\left(\mathbf{\theta},\mathbf{L}_{i}\right)+E\left(\frac{\partial U_{h}\left(\mathbf{\theta},\mathbf{L}_{i}\right)}{\partial\mathbf{\theta}}\right)\frac{1}{\sqrt{n}}\sum_{i=1}^{n} \bar{A}^{-1}S_{i}\right) \\ \\ \approx\frac{1}{n}Var\left(U_{h}\left(\mathbf{\theta},\mathbf{L}_{i}\right)+E\left(\frac{\partial U_{h}\left(\mathbf{\theta},\mathbf{L}_{i}\right)}{\partial\mathbf{\theta}}\right)\bar{A}^{-1}S_{i}\right) \end{matrix}$$

To estimate this **θ** is replaced by its estimate.

With small samples, when asymptotic normality on the risk scale is not obtained, normality on the logit scale can more plausibly be assumed. Calculations on the logit scale also ensure that confidence intervals for the risks do not fall outside the (0,1) bounds. Using the delta method we can derive the variance of $\text{logit}\left(\hat{R}_{h}\right)$:

$$Var\left(\text{logit}\left(\hat{R}_{h}\right)\right)=\sigma_{h}^{2}\left[\frac{\partial\text{logit}\left(R_{h}\right)}{\partial R_{h}}\right]^{2}=\sigma_{h}^{2}\frac{1}{\left(R_{h}\left(1-R_{h}\right)\right)^{2}}$$

**3. Simulation outline**

Input for simulations:

Original Riksstroke data (*Data.orig*) consisting of 18 309 patients in 76 hospitals.

A fixed effects logistic regression model (*Model.orig*) fitted to *Data.orig*.

1. Randomly generate new dichotomous outcomes (*Y*_new_) from a binomial distribution with the fitted values (estimated risk of being dead or dependent at 3 months) from *Model.orig* as parameter.
2. Generate a dataset consisting of the patient specific covariates **X** from *Data.orig* and the new dichotomous outcome variable *Y*_new_.
3. Re-fit the fixed effects logistic regression model to *Y*_new_.
4. Calculate $\hat{Y}\left(h\right)$ using the estimated regression parameters $\hat{\mathbf{\beta}}_{new}$and the estimated hospital effect for hospital *h*, $\hat{\psi}_{h,new}$. $\hat{R}_{sh}$is then calculated by averaging these fitted risks. $\hat{E}\left[Y\right]_{new}=\bar{Y}_{new}$, the sample mean of the new outcome variable.
5. Calculate the lower bound of the *k* × 100% one-sided confidence interval

1. Classify hospital *h* as ‘outlying’ if

,

otherwise as ‘acceptable’. For each hospital this gives 99 classifications.

1. Repeat 1-6 S times.

**4. Checking the variance calculation**

To investigate the accuracy of the asymptotic variance described in section 2 a parametric bootstrap was performed. 1000 simulations were performed and the$\hat{R}_{h}$and asymptotic variance from each repetition were saved. The empirical variance for each hospital was obtained by calculating the variance of the 1000 simulated $\hat{R}_{h}$-values.

As a first check the asymptotic standard deviations for each hospital were summarized in boxplots, and compared to the empirical standard deviations (Figure S1). For all but 9 of the 76 hospitals the empirical standard deviation was within the IQR, 2 hospitals had empirical standard deviations above the third quartile of the asymptotic standard deviations and 7 had empirical standard deviations below the third quartile.

A comparison of the means of the estimated asymptotic standard deviations to the empirical standard deviations shows that on average they are quite similar (Figure S2A). A plot of the difference between the empirical and mean estimated asymptotic standard deviations against hospital size (Figure S2B) shows that the differences tend to be larger for smaller hospitals. The plot of differences vs hospital-specific case-mix adjusted risk does not show a clear pattern, although there is a tendency for hospitals with larger risks (>0.2) to have more negative differences than hospitals with smaller risks (Figure S2C).


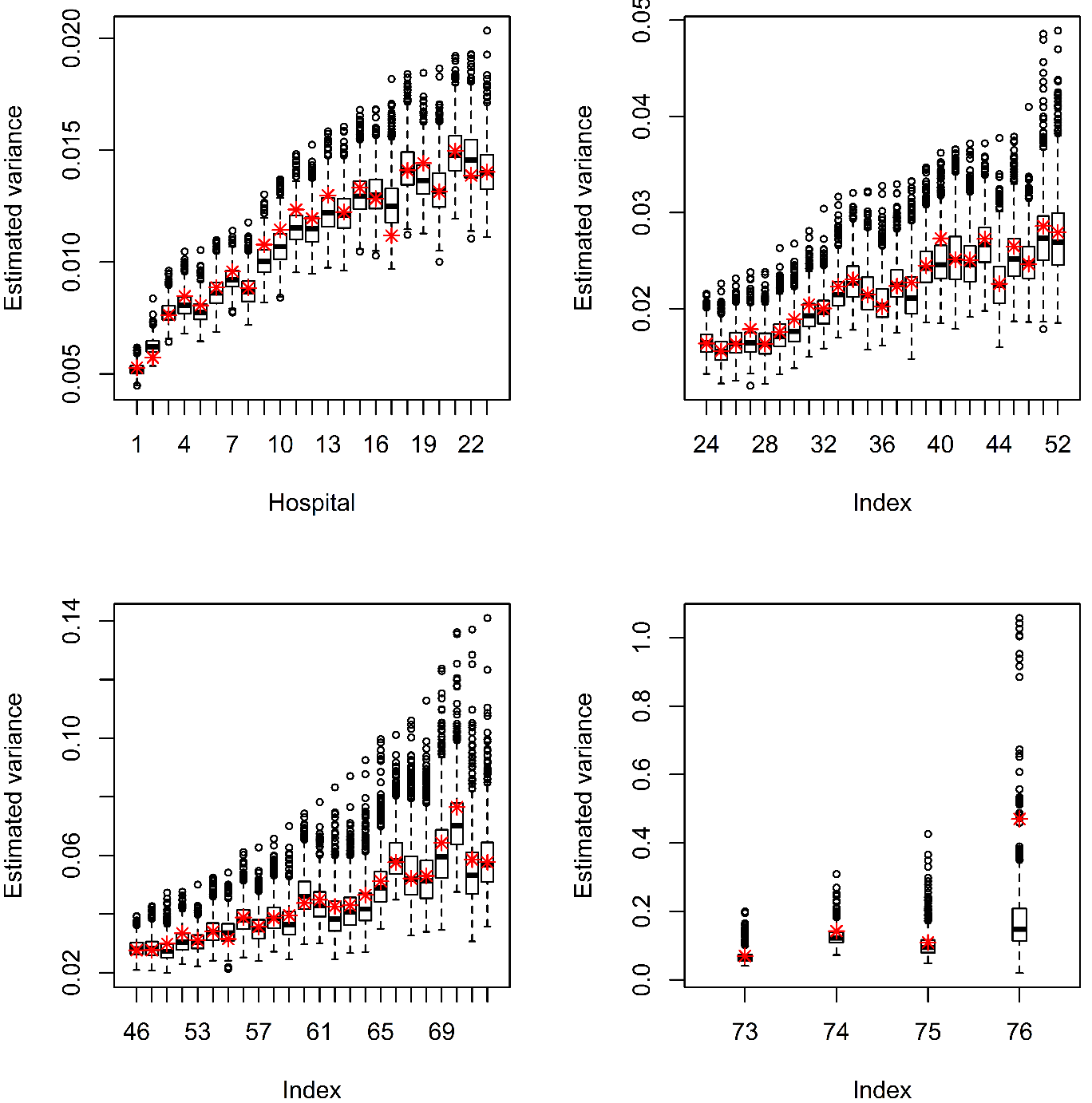


Figure S1: Boxplots of the asymptotic standard deviations for each hospital from 1000 simulations, ordered by the size of the maximum standard deviation. Empirical variances from parametric bootstrap represented by red asterisks.


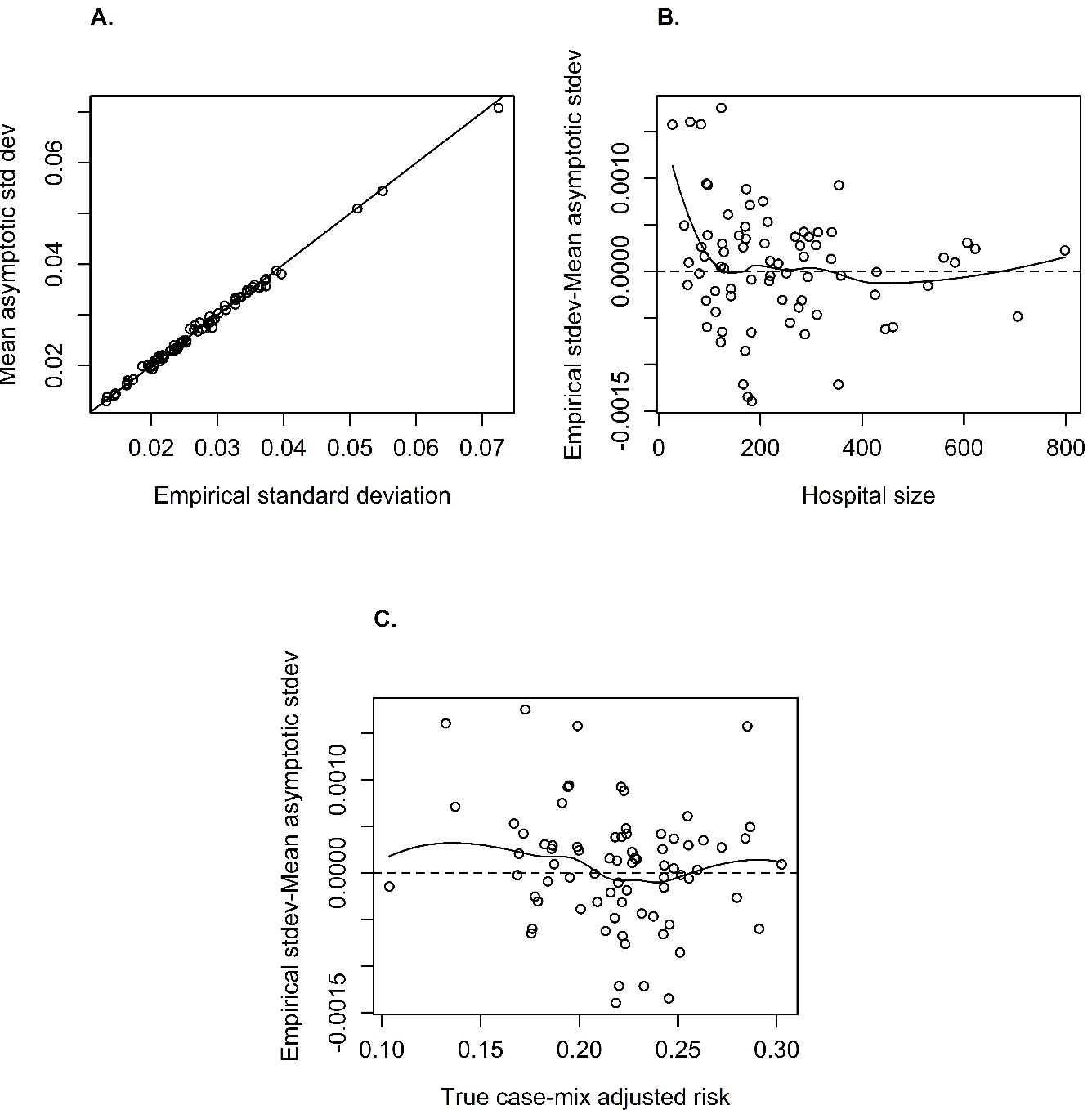


Figure S2: Scatterplots showing A. Mean estimated asymptotic standard deviation vs. empirical standard deviation. B. Difference between theoretical and empirical standard deviation vs hospital size with a fitted smooth curve. C. Difference between theoretical and empirical standard deviation vs hospital-specific case-mix adjusted risks with a fitted smooth curve.

**References**

Varewyck, M., E. Goetghebeur, M. Eriksson and S. Vansteelandt (2014). "On shrinkage and model extrapolation in the evaluation of clinical center performance - Supplementary material." Biostatistics Published online: 8 May 2014.
